# Effects of a Community-Based Behavioral Intervention with a Traditional Atlantic Diet on Cardiometabolic Risk Markers: A Cluster Randomized Controlled Trial (“The GALIAT Study”)

**DOI:** 10.3390/nu13041211

**Published:** 2021-04-07

**Authors:** Mar Calvo-Malvar, Alfonso J. Benítez-Estévez, Juan Sánchez-Castro, Rosaura Leis, Francisco Gude

**Affiliations:** 1 Department of Laboratory Medicine, Complejo Hospitalario Universitario de Santiago de Compostela, 15706 Santiago de Compostela, Spain; mariadelmar.calvo.malvar@sergas.es; 2 Research Methods Group, Health Research Institute of Santiago de Compostela, 15706 Santiago de Compostela, Spain; juanjose.sanchez.castro@sergas.es (J.S.-C.); francisco.gude.sampedro@sergas.es (F.G.); 3 Primary Care Prevention and Health Promotion Network, Carlos III Health Institute, 28029 Madrid, Spain; 4 A Estrada Primary Care Center, A Estrada, 36680 Pontevedra, Spain; 5 Unit of Investigation in Human Nutrition, Growth and Development of Galicia (GALINUT), University of Santiago de Compostela (USC), 15782 Santiago de Compostela, Spain; 6 Unit of Pediatric Gastroenterology, Hepatology and Nutrition, Pediatric Service, University Clinical Hospital of Santiago (CHUS), 15706 Santiago de Compostela, Spain; 7 Pediatric Nutrition Research Group, Institute of Sanitary Research of Santiago de Compostela (IDIS), CHUS-USC, 15706 Santiago de Compostela, Spain; 8 CIBEROBN, (Physiopathology of Obesity and Nutrition) Institute of Health Carlos III (ISCIII), 28029 Madrid, Spain; 9 Clinical Epidemiology and Biostatistics Unit, Complejo Hospitalario Universitario de Santiago de Compostela, 15706 Santiago de Compostela, Spain

**Keywords:** Atlantic diet, nutritional intervention, metabolic risk factors, anthropometric variables, family-based randomized trial, GALIAT Study

## Abstract

The Atlantic diet, the traditional dietary pattern in northern Portugal and northwest Spain, has been related to metabolic health and low ischemic heart disease mortality. The Galiat Study is a randomized controlled trial aimed to assess the effects of the Atlantic diet on anthropometric variables, metabolic profile, and nutritional habits. The dietary intervention was conducted in 250 families (720 adults and children) and performed at a primary care center. Over six months, families randomized to the intervention group received educational sessions, cooking classes, written supporting material, and foods that form part of the Atlantic diet, whereas those randomized to the control group followed their habitual lifestyle. 213 families (92.4%) completed the trial. Adults in the intervention group lost weight as opposed to controls who gained weight (adjusted mean difference −1.1 kg, *p* < 0.001) and total serum cholesterol (adjusted mean difference −5.2 mg/dL, *p* = 0.004). Significant differences in favor of the intervention were found in other anthropometric variables and low-density lipoprotein cholesterol, but changes in triglycerides, high-density lipoprotein cholesterol, inflammation markers, blood pressure, and glucose metabolism were not observed. A family-based nutritional intervention based on the Atlantic diet showed beneficial effects on adiposity and the lipid profile.

## 1. Introduction

Suboptimal diet has been identified as an important risk factor for noncommunicable diseases (NCDs). In the Global Burden Disease Study 2017, 11 million deaths (22% of all deaths among adults) were attributable to dietary risk factors, with non-optimal intake of whole grains, fruits, and sodium accounting for more than 50% of deaths [1]. Suboptimal diet was responsible for more deaths than any other risks globally, including tobacco smoking [1]. The World Health Organization (WHO) Global Action Plan for the Prevention and Control of NCDs 2013–2020 reinforces the need to train the health workforce and strengthen the capacity of health systems, particularly at the primary care level, to address the prevention and control of NCDs [2]. Paradoxically, however, what is a primary prevention problem is being mainly tackled at secondary and even tertiary prevention levels [3]. Although primary care level promotion of a healthy diet and physical activity in adults with no cardiovascular risk factors has shown a positive effect [4], food habits are complex and difficult to modify, and different organizations, such as the WHO [5], the Centers for Disease Control (CDC) [6] or the American Heart Association (AHA) [7] have proposed focusing efforts at different multiple interacting levels (individual, family, community, health policy and management, etc.) involved in health-related behavior [8]. The success of some socially-focused interventions for promoting healthy diets in the population [9,10] suggests that a change is needed in our attempts to prevent and treat obesity and related diseases and that novel interventions should be multisectoral and multidisciplinary in nature, culturally relevant, and community-focused.

The Atlantic diet, the traditional dietary pattern in northwestern Spain and Portugal, is composed, above all, of local, fresh, and seasonal products and involves home cooking and minimally processed foods [11,12]. It has several characteristics in common with the Mediterranean diet such as high consumption of vegetables, fruits, whole grains, and beans, and olive oil as a key fat source. The Atlantic diet is also characterized by high intake of fish and seafood, starch-based products (mainly potatoes and bread), nuts, especially chestnuts, milk and cheese, and moderate consumption of meat and wine. It has been rated as an affordable healthy diet [12,13] and a sustainable diet as defined by the Food and Agriculture Organization (FAO) [14,15]. A recent publication shows that the real consumption pattern in northwestern Spain is far from the traditional and increasingly relevant recommended Atlantic diet [16]. Therefore, a change in the current trends of food consumption towards the recommendations of the Atlantic diet would be beneficial.

A community-focused intervention based on the Atlantic diet and led by a primary health care center may help promoting beneficial changes in food behavior at the population level. The objective of this study was to assess the beneficial effects of such intervention on anthropometric variables, the lipid profile, markers of the metabolic syndrome, and nutrient intake.

## 2. Materials and Methods

### 2.1. Study Design

The GALIAT study (Galicia Atlantic Diet), conducted between March 2014 and May 2015, was approved by the Galician Autonomic Committee for Research Ethics (code 2013/531, approval date 15 January 2014) and registered with clinicaltrials.gov (NCT02391701). The trial was run in accordance with the Declaration of Helsinki and the principles of Good Clinical Practice. Participants were informed of the aims of the study and provided their written consent to be included. For children, signed informed consent was obtained from parents and from children aged >12 years.

The study was a randomized, controlled, parallel-arm, community-focused dietary intervention trial, the protocol of which has been previously reported [11]. The study was performed in the rural town of A Estrada, a municipality in northwestern Spain with a population of 20,700 inhabitants (2017 census). Salient characteristics of the study included the use of the Atlantic diet, a diet congruent with the gastro-cultural heritage of the study area; the family as the intervention unit; fieldwork performed at the primary health care setting; and multilevel actions regarding the dietary intervention with the support of the city hall and other local resources. The duration of the study was six months.

### 2.2. Study Population

**Figure 1:**
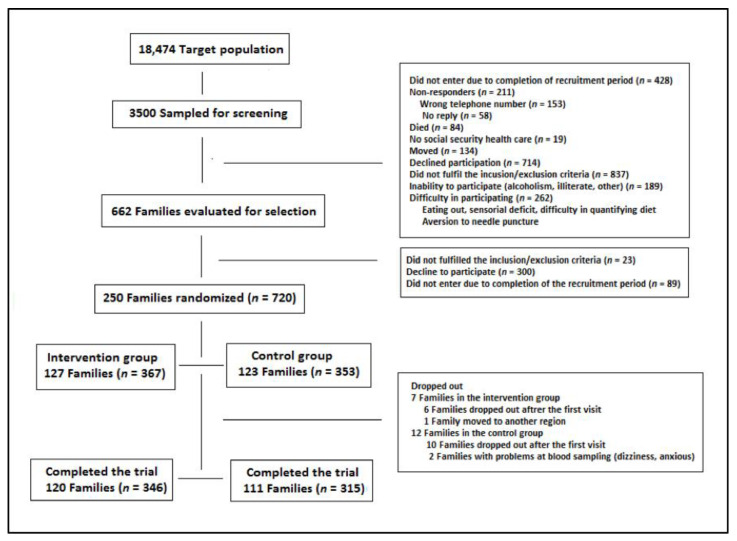
Overview of the study population.

The National Health System Register was used to select a random sample of 3500 persons aged 18–85 years (equally distributed between age groups stratified by decade) from the A Estrada municipality, which constituted the index subjects. Participation in the study was offered to index subjects and their family members who lived in the same household unit. Inclusion criteria for the index subject (male or female) were age 18–85 years, and living in a family unit of at least two members. The other members of the family (either gender) had to be aged 3–85 years. Exclusion criteria for the index subjects were alcoholism, current lipid-lowering treatment, pregnancy, major cardiovascular disease (ischemic heart disease, heart failure, peripheral vascular disease, cerebrovascular disease), dementia, or having a predicted survival of less than one year. The exclusion criteria for the other members of the family were the same except for lipid-lowering treatment. A total of 662 families met the inclusion criteria and were invited to participate, 250 of which (*n* = 720) accepted and gave written consent. Families were randomly allocated (1:1) to the intervention group (127 families, *n* = 367) or to the control group (123 families, *n* = 353) using a computer-generated table of random numbers. Finally, 120 families (*n* = 346) were in the intervention group and 111 (*n* = 315) completed the trial (Figure 1).

### 2.3. Intervention Procedures

The dietary intervention included three nutrition education sessions delivered in the primary health care center and taught by nutritionists aimed to modify food habits in accordance with the characteristics of the Atlantic diet (Table 1). Attendees were providing with supportive material including a recipe book entitled Atlantic Dishes and Menus; a daylong cooking course given by a chef; and delivery of food baskets (free of charge), every three weeks, with a variety of local foods characteristic of the traditional Atlantic diet adapted to the number of family members (Supplementary Table S1).

The recipe book included consensus nutritional recommendations based on the Atlantic diet; daily, weekly, and monthly menu planning, economic menus, and menus adapted to the nutritional needs of children and adults; and recipes based on the Atlantic diet using local products. All the recipes were provided by a chef and adjusted by nutritionists.

In relation to nutrition education sessions, at baseline (visit 0) the nutritionists explained the nutritional recommendations for adults and children, along with information on eating five meals per day without skipping breakfast, on preparing menus, recommended portion sizes, the Atlantic diet, and the food pyramid. They also explained the benefits of physical activity, how to limit sedentarism, and how to use the education material and the recipe book provided. At 3 months, the nutritional recommendations provided were reviewed, food behavior and action plans checked, barriers to progress identified, the changes experienced and the degree of compliance with the recommended diet examined, and doubts answered. At 6 months, a review of progress was made and final messages were given in a 2-h group session in which the researchers and nutritionists again explained the influence of lifestyle on health, how to change to a healthier diet, the importance of physical activity, the characteristics of the traditional Atlantic diet, patterns for designing a healthy diet, and general recommendations on portion sizes.

Fieldwork was performed at a primary health care center, where 20 general practitioners, 3 pediatricians, and 20 nurses took part as collaborating researchers. A further physician, a nurse, and 4 nutritionists were employed to lead the field study at the health care center.

The local city hall provided a van along with personnel to perform the weekly distribution of foodstuffs. A local business provided warehouse space for their storage. A local restaurant lent its facilities for the cooking lessons, and in collaboration with nutritionists, a local hostelry school developed a recipe book and also helped in the cooking lessons. Products included in the food basket were chosen for being part of the Atlantic diet and were donated for free by local food companies.

All personnel involved in fieldwork received theoretical and practical training one month before the study started on how to normalize work procedures. A participatory research approach was followed (research-action-participation) with the aim of promoting mutual learning among researchers, health and city hall workers, and the community (the target).

### 2.4. Outcomes

Outcomes were measured at the individual level. The primary outcome measures were changes at 6 months as compared with baseline of anthropometric variables (weight and body mass index [BMI]) and lipid profile (total cholesterol, high-density lipoprotein cholesterol [HDL-C], and low-density lipoprotein cholesterol [LDL-C]). Secondary outcomes included changes in inflammation markers (C-reactive protein [CRP] and tumor necrosis factor alpha [TNF-α]), glucose and insulin resistance levels (fasting plasma glucose [FPG] and HOMA-IR [homeostatic model assessment of insulin resistance]), and systolic (SBP) and diastolic (DBP) blood pressure.

### 2.5. Study Procedures

Participants were evaluated at baseline (visit 0) and at 3 and 6 months. Data collected at baseline included sociodemographic characteristics; medical history; tobacco and alcohol consumption; medication; anthropometric data; blood pressure; health-related quality of life; adherence to the Atlantic diet; a 3-day food record including two weekdays and either a Saturday or Sunday; and blood sampling for laboratory analysis. Participants in the intervention group received a nutrition education session, a recipe book, a diary with food delivery dates, and a food basket. At 3 months, anthropometric measurements, blood pressure, and laboratory analyses were performed, and the 3-day food record was completed. At 6 months (end of study), procedures were the same as those at baseline.

All laboratory analyses were performed at the Santiago University Hospital. Blood was extracted in the morning following a 10–14 h fast. Total cholesterol, HDL-cholesterol and triglycerides were measured using an Advia 2400 Clinical Chemistry System (Siemens Healthcare Diagnostics), and LDL-cholesterol using the Friedewald formula [17], except when triglycerides were over 400 mg/dL, in which case an enzymatic method was employed with determinations made using the same autoanalyzer. Glucose was measured using Advia 2400 Clinical Chemistry System, and HbA1c via high-resolution liquid chromatography using a Menarini Diagnostics HA-8160 analyzer. CRP, insulin, and IL-6 were determined using an immunometric chemoluminiscence method, employing an Immulite 2000 Immunoassay System. TNF-α was determined in serum using an Immulite 1000 Immunoassay System. Leptin was determined by sandwich ELISA using a DRG Diagnostics kit (Marburg, Germany). All biochemical determinations were made on the day of blood sampling. Blood pressure was measured using an OMRON M3 automatic sphygmomanometer after subjects had been seated for 5 min. All anthropometric measurements were made in triplicate. Each participant was weighed in light clothing to the nearest 0.1 kg using a calibrated beam scale SECA^®^ 701 model-class III, Digital display (Hamburg, Germany). Height was measured without shoes using a portable stadiometer SECA^®^ 213 model (Hamburg, Germany), and recorded to the nearest 0.1 cm. Waist (narrowest point between the bottom of the rib and the top of the iliac crest) and hip (point of greatest prominence of the gluteal muscles) circumferences were measured using a Seca 201 model circumference measuring tape (Hamburg, Germany). Skinfold thickness was measured using a HOLTAIN Tanner/Whitehouse Skinfold caliper (Crosswell, UK). Relative body fat was calculated according to the equation of Siri [18].

Assessment of compliance with recommendations on the Atlantic diet was assessed using a previously validated 14-item Atlantic diet index [19].

Dietary intake was measured using a 3-day food record. Information requested from participants included data on foods consumed (brand names) and characteristics of the methods used for preparation of foods and cooking. Moreover, they were advised to provide the weight of all foods consumed and, when this was not feasible, to use measurements available at home, such as cupfuls and spoonfuls. An equivalency table to assess hand size, household measurement, and weight was given to participants. Nutritionists checked all records completed by the participants. Dietary intake was analyzed using DIAL software [20]. Health-related quality of life was assessed using the Spanish v.2.0 of the Short Form 12 Health Survey (SF-12) [21]. Answers were interpreted with the use of reference values for Spanish populations [22]. The International Physical Activity Questionnaire (IPAQ) (short format) [23] was used to assess physical activity and sedentary behavior, and classified into inactive, minimally active, and active [24]. Details of the study procedures have been described in the study protocol [11] and are summarized in Supplementary Table S2.

### 2.6. Statistical Analysis

Required sample size was calculated for specified serum cholesterol concentration (mean 200 mg/dL, within-subject standard deviation [SD] 36 mg/dL) assuming that the dietary intervention would be associated with a reduction of 10 mg/dL. The sample size required ensuring a minimum predictive power of 80% with a 0.05 type 1 error assuming a 10% drop out rate, and was determined to be 250 families. Sample size and power estimates were calculated using the sample size shop’s GLIMMPSE 2.0 online tool for cluster data (https://glimmpse.samplesizeshop.org/ (accessed on 5 April 2021)). The intention-to-treat (ITT) and the per-protocol (PP) data set were analyzed. The ITT population included all subjects randomized for which data of at least one primary outcome measure was available. The PP population included subjects randomized who completed the study. Multivariate imputation by chained equations method was used to replace missing data. Differences between the intervention and the control group were analyzed using mixed linear models adjusted for age, gender, and baseline values, with the intervention condition deemed a fixed effect and clusters (family) as a random effect. The intraclass correlation coefficient (ICC) was used to assess the correlation of measurements made on individuals of the same cluster (family). Statistical significance was set at *p* < 0.05. The STATA 16 program was used for the analysis of data.

## 3. Results

### 3.1. Baseline Characteristics

Demographic and clinical data of the study subjects at baseline are shown in Table 2. Briefly, all subjects were Caucasian, the mean number of persons per family unit was 2.9, the mean (±SD) subjects’ age was 39.4 ± 20.3 years, and 41% of subjects were males. The mean BMI of adults was 28.0 ± 5.2 kg/m^2^ and the mean total cholesterol level 192 ± 38 mg/dL. There were no statistically significant differences in baseline characteristics between both groups (*p* for all covariates >0.05). Participation in the nutrition education program was high (100% of all families and individuals). A total of 81.7% (300/367) subjects in the intervention arm and 83.3% (294/353) in the control arm filled out completely the three-day food record at baseline at 3 and 6 months.

### 3.2. Anthropometric Variables

Subjects in the intervention arm lost weight (−0.8 kg, 95% confidence interval [CI] −1.1 to −0.5) but subjects in the control arm gained weight (0.4 kg, 95% CI 0.1 to 0.7). The mean difference after adjusting by baseline weight, age, gender, and family cluster was −1.1 kg (95% CI −1.6 to −0.7; *p* < 0.001) in favor of the intervention arm (Table 3). The difference in weight change was higher in male adults than in females or children. The intraclass correlation coefficient (ICC ) was 0.21 revealing an important influence of the cluster (family) on weight loss. There were also statistically significant differences in mean changes of adult BMI (−0.44 kg/m^2^, 95% CI −0.62 to −0.25), the hip/waist ratio, and percentage body in favor of the intervention arm. Results in the per-protocol (PP) dataset were similar (Supplementary Table S3).

### 3.3. Metabolic Measurements

**Figure 2:**
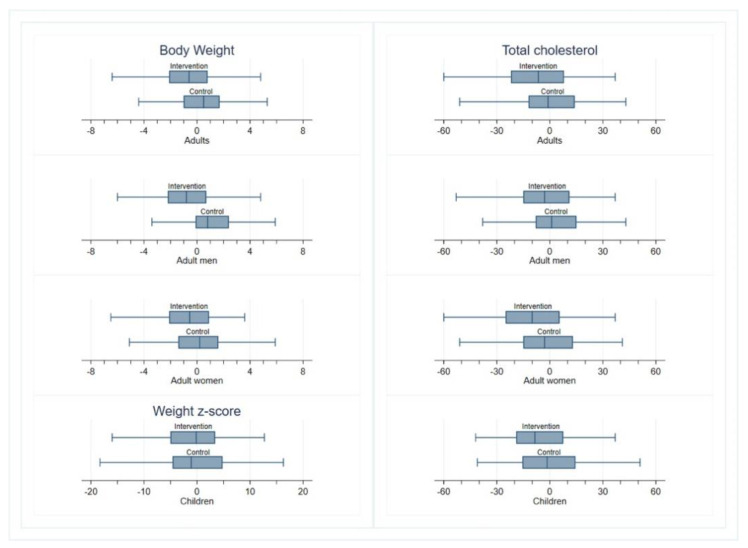
Changes at 6 months in weight and serum cholesterol. Box-and-whisker plots describing the withing-group changes at 6 months in weight and serum cholesterol. The middle line represents the within-group median change, boxes represent the interquartile range (IQR), and whiskers extend to the most extreme observed values with 1.5 × IQR of the nearer quartile.

As shown in Table 3, the difference in the change in total cholesterol between the intervention and control arms at the end of the trial after adjusting for baseline cholesterol, age, gender, and family cluster, was significant at −5.2 mg/dL (95% CI −8.8 to −1.6; *p* = 0.004) in favor of the intervention arm. This difference persisted in the PP analysis (Supplementary Table S3). The difference was greater in woman adults (−5.6 mg/dL, 95%CI −10.6 to −0.6) than in men (−3.6 mg/dL, 95%CI −10.3 to 3.1) or children (−3.7 mg/dL, 95%CI −10.7 to 3.3) (Figure 2). Also, the ICC was 0.22 revealing an important influence of the cluster (family) on the change in total cholesterol levels. A statistically significant difference was also seen in changes of LDL-C levels in favor of the intervention arm, but not in the change in HDL-C or triglycerides. Significant differences between the intervention and control groups in glucose metabolism, inflammatory markers, and blood pressure were not observed (Table 3).

### 3.4. Assessment of Compliance with Recommendations on the Atlantic Diet

Table 4 shows the percentage of participants meeting the Atlantic diet targets and achieving accordance with the recommendations.

The proportion of participants achieving any favorable dietary changes was higher in the intervention than in the control group. It should be noted that in the intervention group there was a greater increase in the consumption of fresh fruits and pulses, and less consumption of sugar-sweetened beverages, than in the control group.

No differences were found in physical activity between both groups.

### 3.5. Dietary Intake

Both the intervention and the control arm subjects showed a significant reduction in mean energy intake by the end of the trial (−215 kcal/day, 95% CI −275 to −154, and −118.4 kcal/day, 95% CI −175.5 to −62.3, respectively) (Table 5). The adjusted analysis showed a significant difference of −152.7 kcal/day (95% CI −242 to −63; *p* = 0.001) in favor of the intervention group. Significant differences between the groups were also seen in the change in the intake of total fat, saturated fatty acids, polyunsaturated fatty acids, monounsaturated fatty acids, and cholesterol. The intervention group subjects also showed a significant increase in their beta carotene and folic acid intakes (Table 5).

Adverse events did not occur in any of the study subjects.

## 4. Discussion

A community-focused, primary health center-led intervention involving the Atlantic diet improved adiposity and the lipid profile through change induced in the family food behavior. The improvements achieved in anthropometric variables were the most relevant. Although the food intake and food composition of subjects in the intervention arm were recorded, the intervention itself placed no restrictions on energy intake. Compared to the control group, subjects in the intervention group experienced a significant reduction in bodyweight of −1.1 kg (−1.7 kg in men and −0.8 kg in women). At the end of the trial, 11% of the adult subjects in the intervention arm had lost 5% of their starting body weight, which is a common criterion for clinically meaningful weight loss [25,26]. A relative reduction of total cholesterol and LDL-cholesterol (−5.2 and −3.4 mg/dL, respectively) among subjects in the intervention arm, particularly in adult women, was also observed. Our findings are similar to those of a systematic review for the US Preventive Services Task Force [27] based on 34 trials conducted in primary care and involving more than 75,000 adults without obesity and no common risk factors for cardiovascular disease (hypertension, dyslipidemia, abnormal blood glucose levels, diabetes). When the results of good quality intervention trials were pooled, significant improvements were seen in adiposity measures (BMI −0.41 kg/m^2^, weight −1.04 kg), and total cholesterol (−2.85 mg/dL), but no evidence was found of any association between behavioral counseling interventions and improvements in HDL-C, triglycerides, or fasting glucose levels.

The reduction in BMI (−0.44 kg/m^2^) in the intervention group as compared to controls is clinically relevant. In a collaborative analysis of 57 prospective studies, which included 894,576 participants from North America and Western Europe, with a BMI between 22.5 and 25 kg/m^2^, it was found that for each BMI increase of 5 kg/m^2^ there was an associated overall mortality 30% higher, ranging from 60–120% mortality for renal, diabetic, and hepatic diseases to 10% mortality for neoplasms [28]. In a meta-analysis of 61 prospective observational studies with almost 900,000 adults, the same group of authors reported that 1 mmol/L lower total cholesterol was associated with about a half lower mortality caused by ischemic heart disease in both sexes at ages 40–49 [29]. Based on our data, a >5% lower risk of ischemic heart disease would have been associated for an estimation linear risk reduction of total cholesterol of −5.2 mg/dL in the intervention group.

Nutrition education sessions based on the traditional diet of the study area were an important part of the intervention. The cultural connection with the culinary traditions of the southern European Atlantic coasts was a positive motivational influence for participants to introduce healthfully dietary changes. Although the Mediterranean diet has been most studied [30,31,32,33], other cultures have similar traditional healthy dietary patterns based on wholesome and nutrient-dense foods [33]. Adherence to the Atlantic diet has been related to good metabolic health and low ischemic heart disease mortality recorded in northern Portugal and northwestern Spain [34,35] and has been promoted in other geographical areas [15]. Healthy, affordable, and sustainable diets that taste good and are culturally appropriate should be promoted for mass dietary change [36] and are one of the strategies for achieving the United Nations Sustainable Development Goals [37].

The methodology of the GALIAT study was based on empowering the community by providing information, building confidence and promoting the flexibility required for healthy food habits to be adopted. Other studies mostly focused on prevention of obesity in children have successfully implemented community-based programs that extend the research of health care delivery systems [9,38,39,40,41,42,43]. Research designs, however, often use quasi-experimental methods and so have weaker internal validity [44]. The high values of the ICC indicate that the cluster (family) factor had an important influence on the food habits established by each family, supporting the effectiveness of family-based approaches for implementing healthful dietary changes.

The strengths of the study were the randomized design and main analyses based on inclusion of all randomized participants, the high retention rates, the use of objective measures, and the recruitment of a random sample representative of the general population. In the context of population reach, we targeted families unselected in terms of health risk and who were representative of Spanish population. We intentionally selected a community of moderate socioeconomic and educational level to strengthen the generalizability of our study. Limitations of the study include a single rural population (of about 20,000 inhabitants), and something that works in small communities may not work in large cities [45]. However, big cities are organized into districts and neighborhoods with their own primary healthcare centers—approaching the conditions of a small town. A further limitation might be contamination bias. Since the study was performed in a small town and became the subject of local and national media interest, some individuals and families may have adopted something of the lifestyle, habits, or food patterns directed at the intervention group. This might have deviated estimates of the effect of the intervention towards the null hypothesis, reducing the differences between the control and intervention arms. In addition, this was a complex intervention and it is not possible to determine which actions of the intervention may have affected the results. Thus, unmeasured or unknown aspects of the intervention, may also exist. The strategy of donating food baskets was an incentive for attendance to educational sessions and to foster adherence. However, this strategy could affect the generalizability of our intervention to populations in which access to or affordability of local, fresh, and seasonal foods might be a barrier. Finally, six months may not have been long enough to properly assess metabolic changes.

In conclusion, the present type of intervention may be useful for improving adiposity and the lipid profile in the general population, with the primary health care center as an appropriate setting for its implementation. The community focus increased the complexity of the intervention by including many non-health care professionals, but it strengthened the degree of social involvement achieved. Although the intervention was performed in a rural Spanish municipality, it might be quite easy to perform in other rural or urban settings.

## Figures and Tables

**Table 1:** Food consumption recommendations of the Atlantic diet.

Food Consumption Recommendations	Servings/Frequency
Bread, cereals, wholegrain cereals, rice, pasta, and potatoes	6–8/day
Olive oil	3–4/day
Fruit	≥3/day
Vegetables	≥2/day
Dairy products	3–4/day
Nuts, preferably chestnuts and walnuts	4–6/week
Fish and seafood	3–4/week
Eggs	3–4/week
Lean meat	3–4/week
Pulses	2–3/week
Fatty meat, cured sausage, margarine, butter	Sparingly/monthly
Sweets, pastries, cakes, ice cream, etc.	Sparingly/monthly

**Table 2:** Baseline characteristics of families and participants (intention-to-treat data set).

Characteristic	Control Arm	Intervention Arm
Families/study subjects, *n*	123/353	127/367
Participants per family, mean ± SD	2.8 ± 1.0	2.9 ± 1.0
Male sex, *n* (%)	142 (48.1)	153 (51.9)
Age, years, mean ± SD	38.6 ± 19.8	40.2 ± 20.8
Marital status ^1^, *n* (%)		
Married/with partner	194 (67.8)	210 (73.4)
Divorced/separated/widowed	33 (11.5)	28 (9.8)
Single	59 (20.6)	48 (16.8)
Educational level ^1^, *n* (%)		
None	29 (10.1)	30 (10.5)
Elementary	120 (41.8)	102 (35.7)
Secondary	91 (31.7)	102 (35.7)
University or higher	47 (16.4)	52 (18.2)
Employment status ^1^, *n* (%)		
Employed	147 (52.1)	137 (48.6)
Retired	40 (14.2)	56 (19.9)
Other	95 (33.7)	89 (31.6)
Smoking status ^1^, *n* (%)		
Never smoker	126 (44.5)	120 (42.0)
Ex-smoker	50 (17.7)	71 (24.8)
Current smoker	107 (37.8)	95 (33.2)
Alcohol intake ^1^, *n* (%)		
Abstainers	123 (43.5)	124 (43.2)
Light drinkers (1–140 g/week)	132 (46.6)	130 (45.3)
Heavy drinkers (>140 g/week)	28 (9.9)	33 (11.5)
Comorbidities ^1^, *n* (%)		
Cardiovascular disease	42 (16.0)	49 (18.3)
Cerebrovascular accident	3 (1.1)	3 (1.1)
Diabetes	16 (5.9)	16 (5.9)
Current medications ^1^, *n* (%)		
Cholesterol-lowering	23 (8.7)	32 (12.5)
Anti-hypertensives	44 (18.1)	56 (24.2)
Health-related quality of life (SF-12v1) ^1^, *n* (%)		
Physical component summary	48.6 (9.3)	47.3 (10.1)
Mental component summary	51.1 (10.1)	52.1 (8.8)
International Physical Activity Questionnaire ^1^, *n* (%)		
Inactive	56 (19.5)	44 (15.4)
Minimally active	68 (23.7)	85 (29.7)
Active	163 (56.8)	157 (54.9)

^1^ Only those aged 18 years or over; SF12v1: 12-item Short Form Health Survey v.1. SD: standard deviation.

**Table 3:** Anthropometric and metabolic variables (intention-to-treat data set).

	Intervention		Control		Adjusted Mean Differences (95% CI)	*p* Value	ICC
	Baseline	6 Months	Baseline	6 Months			
Weight, kg	70.2 ± 22.0	69.8 ± 21.4	67.9 ± 21.2	68.5 ± 21.0	−0.9 (−1.3, −0.5)	<0.001	0.215
Only adults ^1^	77.3 ± 16.9	76.5 ± 16.9	74.8 ± 15.1	75.2 ± 15.2	−1.1 (−1.6, −0.7)	<0.001	-
Women ^1^	71.3 ± 15.8	70.5 ± 15.8	69.2 ± 14.3	69.3 ± 14.3	−0.8 (−1.4, −0.2)	0.012	-
Men ^1^	86.2 ± 14.5	85.3 ± 14.5	83.3 ± 11.9	84.2 ± 11.7	−1.7 (−2.4, −1.0)	<0.001	-
Children ^2^, Z-score	18.0 ± 32.2	17.4 ± 30.4	17.5 ± 30.5	16.5 ± 32.1	0.47 (−2.91, 3.86)	0.783	-
BMI ^1^, kg/m^2^	28.4 ± 5.2	28.1 ± 5.2	27.6 ± 5.1	27.7 ± 5.1	−0.44 (−0.62, −0.25)	<0.001	-
Hip-to-waist ratio	0.91 ± 0.09	0.89 ± 0.10	0.89 ± 0.09	0.89 ± 0.10	−0.012 (−0.019, −0.005)	0.001	0.242
Body fat ^1^, %	35.3 ± 6.9	34.1 ± 7.0	34.3 ± 7.1	33.9 ± 7.1	−0.85 (−1.20, −0.50)	<0.001	-
TC, mg/dL	196 ± 39	189 ± 39	189 ± 36	188 ± 38	−5.2 (−8.8, −1.6)	0.004	0.222
Only adults ^1^	202 ± 39	195 ± 39	196 ± 35	195 ± 36	−4.9 (−9.0, −0.8)	0.020	-
Women ^1^	204 ± 37	194 ± 35	195 ± 33	193 ± 35	−5.6 (−10.6, −0.6)	0.029	-
Men ^1^	199 ± 41	197 ± 44	196 ± 38	198 ± 39	−3.6 (−10.3, 3.1)	0.297	-
Children ^2^	174 ± 32	166 ± 32	158 ± 24	159 ± 27	−3.7 (−10.7, 3.3)	0.305	-
LDL-C, mg/dL	118 ± 34	114 ± 34	112 ± 31	112 ± 32	−3.4 (−6.5, −0.3)	0.034	0.263
HDL-C, mg/dL	55 (47, 66)	55 (46, 65)	55 (46, 65)	54 (45, 64)	−0.9 (−2.2, 0.3)	0.142	0.269
TG, mg/dL	84 (63, 114)	82 (57, 110)	79 (58, 117)	81 (57, 121)	−3.9 (−8.0, 0.5)	0.079	0.282
FPG, mg/dL	86 (79, 93)	83 (77, 90)	84 (79, 93)	82 (76, 90)	0.4 (−1.0, 1.8)	0.563	0.344
HbA1c, %	5.4 (5.2, 5.5)	5.3 (5.2, 5.6)	5.3 (5.2, 5.5)	5.4 (5.2, 5.6)	−0.02 (−0.05, 0.02)	0.343	0.290
Insulin, mIU/L	9.0 (6.1, 13.2)	10.4 (7.4, 14.8)	8.7 (6.0, 12.5)	10.7 (7.4, 14.8)	−0.46 (−1.12, 0.24)	0.189	0.262
HOMA-IR, units	1.89 (1.27, 2.88)	2.16 (1.44, 3.28)	1.86 (1.22, 2.84)	2.18 (1.42, 3.28)	−0.09 (−0.24, 0.08)	0.297	0.297
CPR, mg/L	0.15 (0.06, 0.37)	0.17 (0.08, 0.41)	0.16 (0.06, 0.40)	0.15 (0.06, 0.38)	0.10 (−0.22, 0.42)	0.525	-
TNF-α, mg/dL	7.7 (6.2, 9.8)	6.9 (5.4, 8.7)	8.2 (6.2, 9.9)	7.2 (5.6, 9.0)	−0.18 (−0.52, 0.19)	0.333	0.331
IL-6, pg/mL	2.8 (1.9, 3.9)	2.1 (1.9, 3.0)	2.7 (1.9, 3.7)	2.0 (1.9, 3.1)	−0.01 (−0.17, 0.17)	0.926	0.248
Leptin, ng/mL	7.4 (2.9, 15.7)	5.5 (2.0, 12.0)	6.3 (3.0, 14.0)	5.1 (2.2, 11.4)	−0.29 (−1.22, 0.80)	0.585	0.347
SBP, mmHg	123 ± 18	123 ± 18	122 ± 17	122 ± 17	−0.5 (−2.3, 1.3)	0.590	0.233
DBP, mmHg	71 ± 11	70 ± 10	70 ± 10	69 ± 11	−0.6 (−1.7, 0.6)	0.347	0.146

^1^ ≥18 years of age; ^2^ <18 years or age. Data expressed as mean ± SD or median [interquartile range, 25th–75th percentile]; CI: confidence interval; ICC: intraclass correlation coefficient; BMI, body mass index; TC; total cholesterol; LDL-C, low-density lipoprotein cholesterol; HDL-C, high-density lipoprotein cholesterol; TG, triglycerides; FPG, fasting plasma glucose; HbA1c, glycated hemoglobin; HOMA-IR, homeostasis model assessment insulin resistance; CPR, C-reactive protein; TNF-α, tumor necrosis factor alpha; IL-6; interleukin 6; SBP, systolic blood pressure; DBP, diastolic blood pressure.

**Table 4:** Achievement of Atlantic diet target.

Components	Target	Intervention (*n* = 328)		Control (*n* = 305)	
		Baseline (%) (95% CI)	6 Months (%) (95% CI)	Baseline (%) (95% CI)	6 Months (%) (95% CI)
Bread, cereals, wholegrain cereals, rice, pasta and potatoes	≥6 servings/day	1.93 (0.92, 3.99)	1.21 (0.46, 3.19)	1.44 (0.60, 3.42)	0.98 (0.32, 3.01)
Olive oil	≥3 servings/day	48.76 (43.64, 53.90)	47.88 (42.53, 53.28)	49.57 (44.33, 54.82)	36.07 (30.86, 41.62)
Fresh fruit	≥3 servings/day	43.80 (38.78, 48.96)	50.91 (45.52, 56.28)	42.65 (37.54. 47.92)	36.72 (31.49, 42.28)
Vegetables	≥2 servings/day	7.16 (4.92, 10.32)	6.36 (4.18, 9.57)	6.63 (4.44, 9.78)	3.28 (1.77, 5.99)
Dairy products	≥3 servings/day	70.25 (65.34, 74.73)	75.15 (70.20, 79.52)	67.44 (62.32, 72.17)	64.59 (59.05, 69.76)
Fish and seafood	≥3 servings/week	84.57 (80.48, 87.94)	88.79 (84.90, 91.77)	80.98 (76.50, 84.77)	77.70 (72.68, 82.03)
Lean meat	≥3 servings/week	98.35 (96.37, 99.26)	96.97 (94.46, 98.36)	99.42 (97.7, 99.86)	97.05 (94.42, 98.46)
Eggs	≥3 servings/week	69.70 (64.77, 74.21)	67.58 (62.33, 72.41)	70.03 (64.99, 74.62)	63.93 (58.38, 69.14)
Pulses	≥2 servings/week	22.59 (18.58, 27.18)	44.24 (38.97, 49.65)	21.04 (17.06, 25.65)	32.13 (27.12, 37.58)
Nuts, preferably chestnuts, walnuts, almonds and hazelnuts	≥4 servings/week	8.26 (5.84, 11.58)	9.70 (6.94, 13.40)	4.32 (2.62, 7.05)	5.57 (3.49, 8.79)
Fatty meat, cured sausage, margarine, butter	≤4 servings/month	19.01 (15.29, 23.38)	28.79 (24.15, 33.91)	16.43 (12.89, 20.71)	21.31 (17.07, 26.27)
Sweets, pastries, cakes, candies, ice cream	≤4 servings/month	33.06 (28.41, 38.07)	48.18 (42.83, 53.58)	33.72 (28.93, 38.86)	44.59 (39.10, 50.22)
Sugar-sweetened beverages	≤4 servings/month	54.82 (49.66, 59.88)	67.58 (62.33, 72.41)	48.99 (43.76, 54.25)	53.11 (47.49, 58.66)
Moderate and vigorous physical activity	≥60 min/day	61.71 (56.60, 66.57)	45.45 (40.15, 50.86)	60.52 (55.27, 65.53)	57.70 (52.08, 63.14)

**Table 5:** Differences in dietary intake variables between the intervention and control groups.

	Intervention (*n* = 300)		Controls (*n* = 294)		Adjusted Mean Difference (95% CI)	*p* Value	ICC
	Baseline	6 Months	Baseline	6 Months			
Total energy, kcal/day	1945 ± 534	1730 ± 478	2026 ± 563	1907 ± 528	−152.7 (−242.0, −63.4)	0.001	0.374
Protein, % E	16.2 ±2.8	17.6 ± 3.0	16.0 ± 2.6	16.9 ± 3.0	0.62 (−0.02, 1.26)	0.060	0.471
Total fat, % E	41.2 ± 6.5	39.5 ± 7.2	41.3 ± 6.8	41.4 ± 7.2	−1.95 (−3.52, −0.37)	0.016	0.537
Monounsaturated fatty acids, g/day	43.1 ± 14.9	36.6 ± 13.0	45.1 ± 14.6	41.9 ± 15.4	−5.10 (−7.87, −2.33)	<0.001	0.417
Polyunsaturated fatty acids, g/day	10.8 ± 4.1	9.7 ± 4.5	11.3 ± 4.6	10.6 ± 4.5	−0.89 (−1.81, 0.03)	0.058	0.423
Saturated fatty acids, g/day	27.9 ± 11.4	23.1 ± 8.9	29.1 ± 11.4	28.1 ± 10.6	−4.47 (−6.22, −2.72)	<0.001	0.353
Cholesterol, g/day	307 ± 132	269 ± 104	325 ± 151	301 ± 115	−32.4 (−54.4, −10.3)	<0.001	0.401
Total carbohydrates, % E	48.9 ± 8.6	50.2 ± 9.6	50.0 ± 9.4	49.7 ± 9.3	0.96 (−1.07, 3.00)	0.354	0.516
Solvable fiber, g/day	4.3 ± 1.6	4.1 ± 1.8	4.2 ± 2.0	3.9 ± 1.7	0.19 (−0.14, 0.52)	0.260	0.474
Unsolvable fiber, g/day	7.2 ± 3.0	7.4 ± 3.8	6.9 ± 3.6	6.5 ± 3.4	0.65 (−0.00, 1.31)	0.050	0.477
Starch, g/day	96 ± 38	87 ± 32	106 ± 42	99 ± 38	−9.0 (−15.6, −2.3)	0.009	0.430
Calcium, mg/day	840 ± 313	812 ± 302	806 ± 308	788 ± 296	1.30 (−50.1, 47.5)	0.958	0.331
Iron, mg/day	13.6 7.0	13.5 ± 6.1	13.6 ± 6.1	13.0 ± 5.7	0.48 (−0.51, 1.46)	0.346	0.260
Ascorbic acid, mg/day	118 59	124 ± 62	114 ± 61	105 ± 62	15.9 (3.2, 28.7)	0.014	0.517
Vitamin E, mg/day	7.4 ± 3.0	7.3 ± 3.5	7.4 ± 3.6	6.9 ± 3.4	0.26 (−0.46, 0.98)	0.472	0.489
Beta carotene, µg/day	1856 (1115, 2899)	2457 (1490, 4169)	1794 (1011, 2726)	1426 (637, 2389)	1053 (626, 1581)	<0.001	0.538
Folic acid, µg/day	218 (81)	235 (89)	207 (75)	206 (75)	23.8 (6.9, 40.7)	0.006	0.536
Vitamin B12, µg/day	4.7 (3.7, 6.6)	4.6 (3.3, 6.3)	4.7 (3.6, 7.0)	4.4 (3.4, 5.9)	−0.03 (−0.53, 0.52)	0.899	0.466
Vitamin B6, g/day	2.0 ± 0.8	2.1 ± 0.7	2.1 ± 0.8	2.1 ± 0.8	−0.01 (−0.16, 0.14)	0.896	0.370

Data expressed as mean ± SD or median [interquartile range, 25th–75th percentile]; CI: confidence interval; ICC: intraclass correlation coefficient; E: energy intake.

## Data Availability

The datasets generated and analyzed during the current study will be made available through a publicly accessible repository on publication, at the Runa Digital Repository (runa.sergas.gal). To gain access, data requestors will need to sign a data access agreement. Proposals should be directed to alfonsojavier.benitez.estevez@sergas.es and/or francisco.gude.sampedro@sergas.es.

## Supplementary Materials

The following are available online at https://www.mdpi.com/article/10.3390/nu13041211/s1. Table S1: Food provided per subject in the food basket, Table S2: Details of the study procedures, Table S3: Anthropometric and metabolic variables (per-protocol data set).

## Abbreviations

BMI, body mass index; CI, confidence interval; CRP, C-reactive protein; DCCT, Diabetes control and complications trial; DBP, diastolic blood pressure; FFQ, food frequency questionnaire; FPG, fasting plasma glucose; HDL-C, high-density lipoprotein cholesterol; HOMA-IR, homeostatic model assessment of insulin resistance; ICC, intraclass correlation coefficient; ITT, intention-to-treat; LDL-C, low-density lipoprotein cholesterol; MET, metabolic equivalent task; NCDs, noncommunicable diseases; PP, per-protocol; SBP, systolic blood pressure; TC, total cholesterol; TNF-α, tumor necrosis factor alpha.
