# Supplementary material for: Effects of a Community-Based Behavioral Intervention with a Traditional Atlantic Diet on Cardiometabolic Risk Markers: A Cluster Randomized Controlled Trial (“The GALIAT Study”)
Source: Nutrients. 2021 Apr 7;13(4):1211. doi: 10.3390/nu13041211 (PMC8067574; doi:10.3390/nu13041211)
Supplement: Supplementary file 1 [file nutrients-13-01211-s001.zip › Supplementary Table S1 .pdf]

Supplementary Table S1. Food provided per subject in the food basket

Turnip greens	250 g/week
Cabbage	200 g/week
Mushrooms	64 g/week
Tomatoes	277 g/week
Zaragallada [1]	175 g/week
Mirabelle plums	140 g/week
Mussels	57 g/week
Low fat cheese	100 g/week
Olive oil	339 g/week
White wineb	250 mL/week
Red wine [2]	250 mL/week

[1] A tomato, green pepper and onion sauce used in Galicia to help fill samosa-like pies.

[2] Wine was provided only to family members who were not teetotalers.
