# Supplementary material for: Effects of a Community-Based Behavioral Intervention with a Traditional Atlantic Diet on Cardiometabolic Risk Markers: A Cluster Randomized Controlled Trial (“The GALIAT Study”)
Source: Nutrients. 2021 Apr 7;13(4):1211. doi: 10.3390/nu13041211 (PMC8067574; doi:10.3390/nu13041211)
Supplement: Supplementary file 1 [file nutrients-13-01211-s001.zip › Supplementary Table S2.pdf]

Supplementary Table S2. Details of the study procedures

Time line	Intervention	Control
Pre- assessment	A B C	A B C
Basal (time 0)	D E F G<br>Randomization<br>H I J<br>K L M	D E F G<br>Randomization<br>H I J
1 week<br>4 weeks<br>7 weeks<br>10 weeks	O<br>O P<br>O<br>O	
3 months	F G L	F G
13 weeks<br>16 weeks<br>19 weeks<br>22 weeks	O<br>O<br>O<br>O	
6 months	F G I J<br>L	F G I J<br>L K
	Measurement of outcomes	

A	Interview between index subject and physician at the health centre. Verbal explanation of the project
B	Provision of written explanation of the project and consent forms (for children/adults/parents & guardians)
C	If participation is accepted, appointment made to see medical team, with family, for baseline assessment, and to provide instructions regarding blood extraction
D	Check family members meet inclusion criteria but no exclusion criterion

E	Collect consent forms
F	Blood extraction
G	Anthropometric measurements, blood pressure
H	Complete questionnaire on socioeconomic status, check personal and family medical history
I	Complete questionnaire on health-related quality of life, physical activity, sedentarism, diet quality, food consumption patterns, food frequency, alcohol consumption, use of tobacco
J	Hand out of 3-day food record forms
K	Distribute book with educational material and recipes
L	Nutrition education course (30-40 min) provided to individual families by nutritionists; recommendations for adults and children; information on eating five meals per day; preparing menus; explain the Atlantic diet and food pyramid; benefits of physical activity; how to limit sedentary activity; how to use the education material provided
M	Diary with food delivery dates (every 3 weeks)
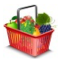	Begin delivery of food packages (adapted for the number of family members)
O	Complete questionnaire on food habits, consumption of delivered foods, physical activity/sedentarism, answering any questions
P	Group session (2 h) by researchers and nutritionists to explain influence of lifestyle on health, changing to a healthier diet, importance of physical activity, characteristics of the traditional Atlantic diet, patterns for designing a healthy diet
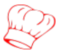	Cooking class led by teaching chefs; recommendations on portion size
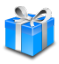	Gift of food basket, educational material and recipe book
