# Supplementary material for: Effects of a Community-Based Behavioral Intervention with a Traditional Atlantic Diet on Cardiometabolic Risk Markers: A Cluster Randomized Controlled Trial (“The GALIAT Study”)
Source: Nutrients. 2021 Apr 7;13(4):1211. doi: 10.3390/nu13041211 (PMC8067574; doi:10.3390/nu13041211)
Supplement: Supplementary file 1 [file nutrients-13-01211-s001.zip › Supplementary Table S3 .pdf]

Supplementary Table S3. Anthropometric and metabolic variables (per-protocol data set)

	Intervention (*n* = 346)		Control (*n* = 315)		Adjusted mean differences (95% CI)	*P* value	ICC
	Baseline	6 months	Baseline	6 months			
Weight, kg	70.2 ± 22.0	70.4 ± 21.4	67.6 ± 21.7	68.2 ± 21.4	-0.9 (-1.3, -0.5)	<0.001	0.079
Only adults[1]	77.4 ± 17.0	76.6 ± 17.0	75.0 ± 14.9	75.5 ± 15.0	-1.2 (-1.7, -0.7)	<0.001	-
Women[1]	71.4 ± 16.0	70.7 ± 16.0	69.7 ± 14.1	69.8 ± 14.1	-0.8 (-1.4, -0.2)	0.011	-
Men[1]	86.3 ± 14.5	85.5 ± 14.5	83.1 ± 12.3	84.1 ± 12.0	-1.7 (-2.4, -1.0)	<0.001	-
Children[2] Z-score	18.5 ± 3.8	18.0 ± 3.6	17.5 ± 3.8	16.4 ± 4.0	0.69 (-2.97, 4.35)	0.689	-
BMI[1], kg/m^2	28.5 ± 5.3	28.2 ± 5.2	27.7 ± 5.1	27.9 ± 5.0	-0.45 (-0.63, -0.27)	<0.001	-
Hip-to-waist ratio	0.91 ± 0.09	0.89 ± 0.10	0.89 ± 0.09	0.89 ± 0.10	-0.013 (-0.020, -0.006)	<0.001	0.108
Body fat[1]	35.4 ± 6.9	34.0 ± 7.0	34.4 ± 7.1	34.1 ± 7.0	-0.88 (-1.22, -0.53)	<0.001	-
TC, mg/dL	195 ± 38	188 ± 38	190 ± 36	189 ± 36	-5.3 (-8.9, -1.8)	0.003	0.086
Only adults[1]	201 ± 37	195 ± 39	197 ± 34	196 ± 35	-5.0 (-9.1, -0.9)	0.017	-
Women[1]	202 ± 34	193 ± 33	197 ± 32	195 ± 33	-6.0 (-11.1, -1.0)	0.018	-
Men[1]	199 ± 41	197 ± 43	197 ± 38	198 ± 38	-3.2 (-9.8, 3.5)	0.354	-
Children[2]	170 ± 28	162 ± 28	160 ± 24	159 ± 25	-3.4 (-10.1, 3.2)	0.307	-
LDL-C, mg/dL	117 ± 33	114 ± 33	113 ± 31	113 ± 31	-3.6 (-6.8, -0.4)	0.025	0.127
HDL-C, mg/dL	55 [47, 66]	54 [46, 65]	56 [46, 65]	55 [45, 65]	-1.0 (-2.2, 0.3)	0.312	0.136
TG, mg/dL	84 [63, 114]	83 [57, 110]	80 [59, 120]	82 [57, 125]	-3.7 (-7.8, 0.6)	0.089	0.145
FPG, mg/dL	86 [79, 93]	83 [77, 90]	84 [78, 93]	81 [76, 90]	-1.0 (-2.2, 0.3)	0.132	0.136
HbA1c	5.4 [5.2, 5.5]	5.4 [5.2, 5.6]	5.3 [5.1, 5.6]	5.4 [5.2, 5.6]	-0.02 (-0.05, 0.02)	0.298	0.187
Insulin, mIU/L	9.1 [6.0, 13.2]	10.4 [7.5, 14.9]	8.7 [6.0, 12.4]	10.7 [7.3, 14.8]	-0.43 (-1.09, 0.28)	0.230	0.128
HOMA-IR, units	1.91 [1.27, 2.92]	2.17 [1.46, 3.32]	1.86 [1.20, 2.83]	2.19 [1.40, 3.29]	-0.08 (-0.24, 0.00)	0.324	0.171
CPR, mg/L	0.14 [0.06, 0.36]	0.18 [0.08, 0.46]	0.18 [0.07, 0.45]	0.18 [0.06, 0.50]	-0.00 (-0.28, 0.28)	0.999	-
TNF-α, mg/dL	7.6 [6.1, 9.8]	6.8 [5.4, 8.7]	8.3 [6.4, 10.0]	7.3 [5.7, 9.1]	-0.23 (-0.57, 0.14)	0.215	0.223
IL-6, pg/mL	2.7 [1.9, 3.9]	2.1 [1.9, 3.0]	2.7 [1.9, 3.7]	1.9 [1.9, 3.0]	-0.02 (-0.18, 0.16)	0.854	0.107
Leptin, ng/mL	7.7 [2.9, 15.9]	5.6 [1.9, 12.2]	6.4 [2.9, 14.5]	5.2 [2.1, 11.9]	-0.22 (-1.18, 0.89)	0.682	0.255
SBP, mmHg	124 ± 18	123 ± 18	123 ± 17	122 ± 18	-0.5 (-2.3, 1.3)	0.572	0.097
DBP, mmHg	71 ± 11	70 ± 10	70 ± 10	70 ± 10	-0.5 (-1.7, 0.6)	0.356	0.038

[1] ≥ 18 years of age; [2] < 18 years or age. Data expressed as mean ± SD or median [interquartile range, 25th-75th percentile]; CI: confidence interval; ICC: intraclass correlation coefficient; BMI, body mass index; TC; total cholesterol; LDL-C, low-density lipoprotein cholesterol; HDL-C, high-density lipoprotein cholesterol; TG, triglycerides; FPG, fasting plasma glucose; HbA1c, glycated hemoglobin; HOMA-IR, homeostasis model assessment insulin resistance; CPR, C-reactive protein; TNF-α, tumor necrosis factor alpha; IL-6; interleukin 6; SBP, systolic blood pressure; DBP, diastolic blood pressure.
